# Bacterial DNA Contamination of Commercial PCR Enzymes: Considerations for Microbiome Protocols and Analysis

**DOI:** 10.3390/microorganisms13040732

**Published:** 2025-03-25

**Authors:** Andrew M. Skidmore, Steven B. Bradfute

**Affiliations:** Center for Global Health, Department of Internal Medicine, University of New Mexico Health Sciences Center, Albuquerque, NM 87131, USA; amskidmore@salud.unm.edu

**Keywords:** microbiome, bacterial sequencing, contamination, environmental DNA

## Abstract

The microbiome remains a top area of research, and it is now common to examine any organic and inorganic samples for bacterial colonization. However, due to the ubiquity of bacteria in the environment, separating the low-burden colonization of bacteria from the possible contamination of laboratory reagents remains problematic. When examining samples of expected low bacterial burden, it is common to first amplify any bacterial DNA present through PCR before sequencing. In this work, we examined nine different commercial PCR enzymes and their reaction components as possible sources of bacterial DNA contamination. We found contaminating bacterial DNA in seven of the nine reactions, and this DNA was shown to come from a variety of species. Importantly, we were able to perform these studies solely with endpoint PCR and Sanger sequencing, which are more accessible and affordable than high-throughput, short-read sequencing and real-time PCR. This work confirms that there needs to be an increased emphasis on including control reactions in microbiome studies so that contaminating DNA sequences can be identified and addressed, and that this can be achieved with minimal resources.

## 1. Introduction

Due to its stability and ubiquity in the environment, the DNA contamination of laboratory consumables and reagents is nearly inevitable. This is of particular concern with highly sensitive analysis methods, such as polymerase chain reaction (PCR) assays and next-generation sequencing. Nucleic acid contamination has been a particular problem in studies of human microbiomes [1,2,3,4,5,6]. Due to this, controversy has arisen as to whether some tissues are sterile, as was previously assumed, or if they have low-level bacterial communities, such as the human placenta [1,3]. This has led to the coining of the term “kitome” or contaminating bacterial sequences that result from laboratory consumables and nucleic acid isolation kits [3,5]. It has also been reported that PCR materials can be sources of DNA contamination [1,7], which was proven by treatment of PCR master mixes with DNases that specifically target double-stranded DNA, which have recently become commercially available [7,8]. Currently, the best practice when examining samples expected to have low bacterial burden is to include a variety of negative control samples to exclude those sequences that are likely due to contamination [2,3,6]. Despite supposed widespread knowledge of the issue of contamination, only a minority of microbiome papers report using methodology to control for it, and most studies lack any particular negative controls [9]. However, the inclusion of additional control reactions in short-read sequencing protocols and analysis is resource intensive and can require significant additional computational time. This makes arguing in favor of these controls difficult, especially in the case of researchers with less resources available to them.

While many researchers have moved away from using amplicon-based sequencing to determine the makeup of the microbiome in favor of metagenomics processes, PCR remains an important step in the characterization of the microbial communities of low-burden samples, and amplicon-based approaches still have many advantages [10,11].

A variety of bioinformatic tools are available and in development to control for bacterial contamination [12,13,14]. However, many of these tools are overly blunt and only remove samples of low abundance, or assume that any sequence that is present in a negative control must be a contaminant in an experimental reaction [13]. Thus, there is still a need for experimental validation and human oversight of these filtering processes. These tools need further development, and they still function best when used in conjunction with thorough negative sampling.

Here, we present data that show that multiple commercially available PCR enzymes and their components are contaminated with different dominant bacterial DNAs, and that this contaminating DNA can be detected with endpoint PCR and Sanger sequencing. Our data indicate that DNA contamination should be examined regardless of the molecular biology reagents used, and that this can be carried out rapidly, for minimal cost, and without short-read sequencing, which makes the large number of control reactions needed feasible for labs with less access to resources. These contaminants can then be excluded or otherwise addressed in further analysis of the microbiome data.

## 2. Materials and Methods

### 2.1. PCR

Nine PCR enzymes (1–9) were obtained from five manufacturers; the identities of these enzymes and manufacturers can be found in Supplementary Table S1. Reactions were carried out according to manufacturer’s recommendations. To test for bacterial DNA contamination, two sets of reactions were performed per enzyme. First, *E. coli* DNA was used as a template to confirm the accuracy of the primer set for bacterial DNA detection as well as to ensure that reactions were running as expected. Second, to determine if contamination was present, reactions were run with no DNA template and instead used water only. Primers were obtained from Invitrogen. Positive control reactions contained DNA extracted from overnight cultures of *E. coli* isolated from a human fecal sample, a generous gift from Dr. Julie In. Primers and reaction conditions were adapted from published studies [15,16]. PCRs were prepared under laminar flow, in hoods that are only used for PCR preparation, using aseptic technique. Information regarding the enzyme manufacturer, deoxynucleotide triphosphate (dNTP) mix aliquot, water aliquot used, and if the enzyme was premixed is listed in Table 1. All reactions were performed using Invitrogen RT-PCR grade water except for enzyme 9, which came with its own molecular-grade water. All PCR cycling conditions can be found in Table 2, Table 3, Table 4, Table 5 and Table 6. Primer sequences can be found in Table 7.

### 2.2. Gel Electrophoresis

Five μL of PCR product was combined with 1 μL of 6X gel loading dye and then separated by gel electrophoresis in a 1% agarose gel using the Owl system from ThermoFisher. Gels were cast using premixed SYBRsafe 0.5% solution (ThermoFisher, Waltham, MA, USA) and run in 1X TBE buffer. Gel images were developed using ultraviolet light in a UVP UVsolo touch system (Analytikjena, Jena, Germany).

### 2.3. Size Selection of Contaminating Bands for Sequencing

Samples were mixed with 10X loading dye (ThermoFisher) and separated by electrophoresis in the EGel system (ThermoFisher) using clone well 0.8% and Size Select 2% agarose gels according to manufacturer’s directions (ThermoFisher). Bands were selected for sequencing at 500 bases, the expected size of the V3-4 region of the bacterial 16S rRNA. One enzyme generated bands at 1000 bases that were also collected. Bands found at approximately 100 bases were collected if present. Samples were collected in nuclease free water.

### 2.4. Sanger Sequencing of Samples

Samples were submitted to GENEWIZ (Azenta, Burlington, MA, USA). Samples were sequenced in separate forward and reverse reactions using the same primers from the PCRs.

### 2.5. Informatics Analysis of Sample Sequences

Chromatogram files, the raw spectral files that are used to examine the Sanger sequencing results of the sequences, were used for quality control. Read ends were trimmed to remove long runs of unidentified bases, and the first and last included base had to have a quality score greater than or equal to 20. After trimming, the sequences were used to generate a phylogenetic tree using Mega 11 software [17]. The greatest likelihood algorithm was used, and 1000 bootstraps were performed. The tree file was visualized using the Interactive Tree of Life (ITOL) for color coding and labeling [18]. Trimmed sequences were used to query the NCBI GenBank database via megablast, searching for highly similar nucleotide sequences. The top three hits from each search were identified and genus, and species if present, were recorded as well as the percent coverage and percent identity of each read. The similarity between samples was calculated as percent identity using Clustal Omega [19].

## 3. Results

### 3.1. Seven of Nine Commercially Available PCR Enzymes Were Found to Have Bacterial DNA Contamination

**Figure 1:**
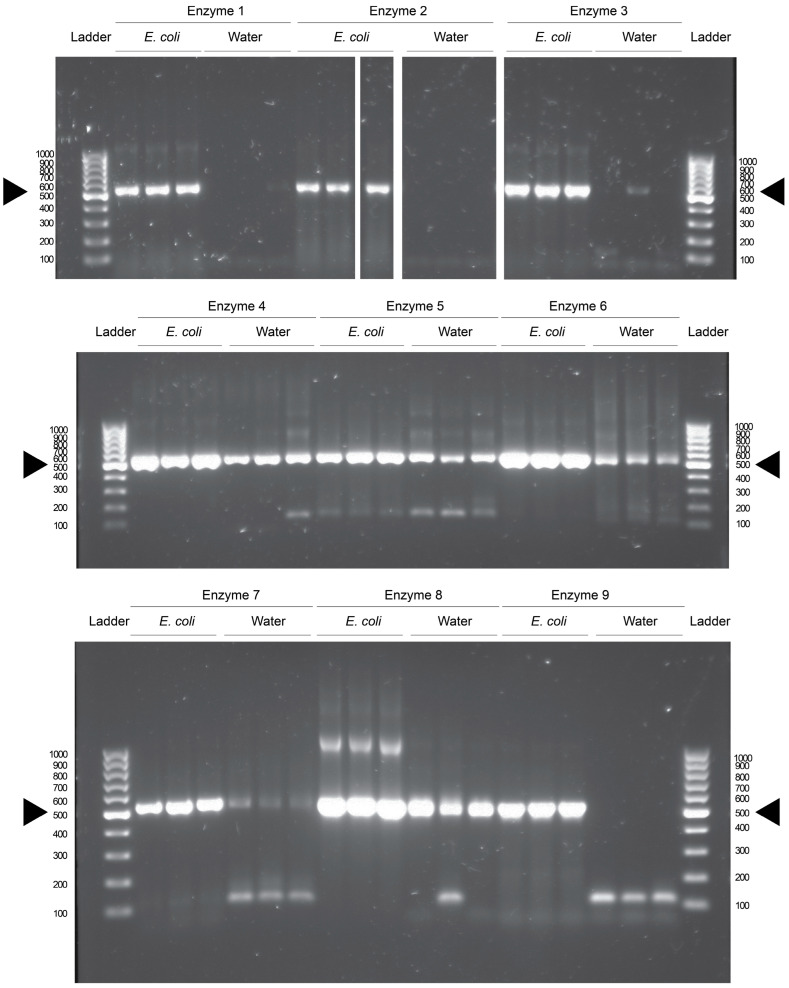
The detection of contaminating bacterial DNA in PCR enzymes. A broad sampling of PCR enzymes reveals that many are contaminated with bacterial DNA. Representative gel images of nine PCR enzymes from 6 manufacturers reveal that for 7 of these enzymes, all except enzymes 2 and 9, there is contaminating DNA, resulting in the production of DNA product of the target size. Primers targeted the V3-4 region of the bacterial 16S rRNA gene. As shown in *E. coli* DNA control reactions, this product comprises approximately 500 bases, indicated by black arrows. DNA contamination is unlikely to have been introduced in our lab, as not every reaction is contaminated, and contamination is consistent across technical replicates. The ladder is the molecular size maker, labeled as the number of bases per band. *E. coli* labeled reactions contain *E. coli* DNA; water reactions contain no exogenous DNA. Images are from a single representative experiment with three technical replicates per reaction. Each lane is a single technical replicate of the indicated condition.

Commercially available DNA polymerases and their reaction components were purchased from five manufacturers, and nine polymerase products were tested for bacterial DNA contamination in triplicate using *E. coli* DNA as a template or water as a negative control. One of the polymerases was premixed, and all kits provided all necessary components except the dNTP solution, which was shared between the reactions as indicated in Table 1. The polymerases were grouped into similar conditions and the reactions were run in groups in a thermocycler. The PCR products were then visualized after gel electrophoresis using UV light. It was found that seven of the nine polymerases had bands of equivalent size to the V3-4 region of the 16S rRNA in water control reactions (Figure 1). This is seen when comparing the reactions to positive control reactions which contained template DNA isolated from overnight cultures of *E. coli* (Figure 1). These data indicate that bacterial DNA contamination is present in seven of the nine commercially available products. Enzyme and manufacturer identities are found in Supplementary Table S1.

These results are not due to contamination from our laboratory, as two of the enzymes (2 and 9) do not have visible contamination after PCR. Enzyme 1 also had low levels of contamination; however, it does have a faint band in the third technical replicate of the water reaction, indicating a small amount of DNA contamination. The enzymes that display contamination were prepped at different times, and run in different reactions in the thermocycler, reducing the likelihood of cross-contamination between different reactions.

While DNA gels have become less popular as an endpoint for the detection of DNA, they are a highly sensitive assay. A DNA band can be visualized from as little as 2 ng/μL of total concentration of DNA in a solution. Due to the exponential nature of the PCR, this amount of DNA can be generated from sub-picogram amounts of DNA in the initial reaction. Thus, the blank lanes in the gel indicate samples that were free from any DNA that could be amplified by the selected primer set. If there was DNA present, but not enough to generate the required concentration visualizable in a gel, it would not be apparent here.

It is possible that there would be detectable reads during a next-generation sequencing run, as there have been reports of contamination appearing at levels of 100 s of reads per lane [5]. However, that would then open the possibility that this contamination occurred during library preparation, or during chip loading and sequencing itself, which would be out of our control. For a truly comprehensive approach, the size selection of PCR products could be performed for the approximately 500 base-pair contaminants, even in those reactions that do not have visible DNA on an agarose gel. These selected products could then be submitted for Sanger sequencing. However, this is unlikely to generate useful sequencing data, as the ideal amount of DNA for a Sanger read is roughly equivalent to the limit of detection in an agarose gel [20,21].

### 3.2. DNA Contamination Derived from Different Bacterial Sources

PCR products from these reactions were submitted for Sanger sequencing using either the forward or reverse primer from the PCR. Each contaminating band of the expected size of the V3-4 regions (500 bp) was selected, as were bands of 100 bp if they were present and distinct after electrophoresis. Enzyme 8 also produced bands of 1000 bp in its positive control reactions, and these were selected as well. The chromatograms were used to trim low-quality ends, and any sequences that did not result in usable reads were excluded. These sequences were then used to determine (a) if there was an association between the contaminating DNA and the polymerases used in each reaction and (b) the most likely identity of the contaminating bacteria.

**Figure 2:**
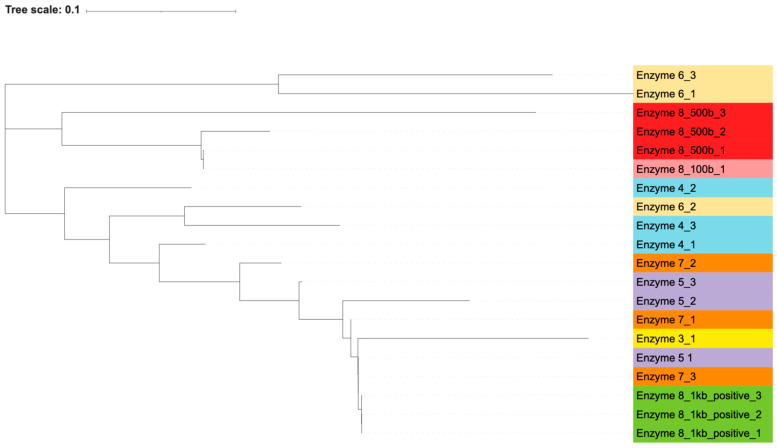
Highest-likelihood phylogeny of forward sequences. A phylogenetic tree demonstrating the highest-likelihood similarity between contaminating bacterial sequences from various PCR enzymes using the forward primer for sequencing. Contaminating PCR sequences that occurred in the negative-control water reactions were submitted for Sanger sequencing by the company GENEWIZ. Three sequences from *E. coli* reactions were also included from enzyme 8, as they were double the expected size. All sequences were of the 500 base expected size unless otherwise indicated. This tree demonstrates that the most significant sorting factor of these sequences was the enzyme that was used for DNA synthesis. This shows that these contaminating sequences were likely present in the PCR components themselves and were not introduced by our lab or in materials that were already present in our lab such as the water, or dNTP mix. The tree was generated using a maximum likelihood algorithm with 1000 bootstrap replicates. Alignment was performed using the Clustal algorithm in the MEGA11 software package. The tree was labeled and visualized using Interactive Tree of Life. Colors are identical for technical replicates of the same enzyme.

**Figure 3:**
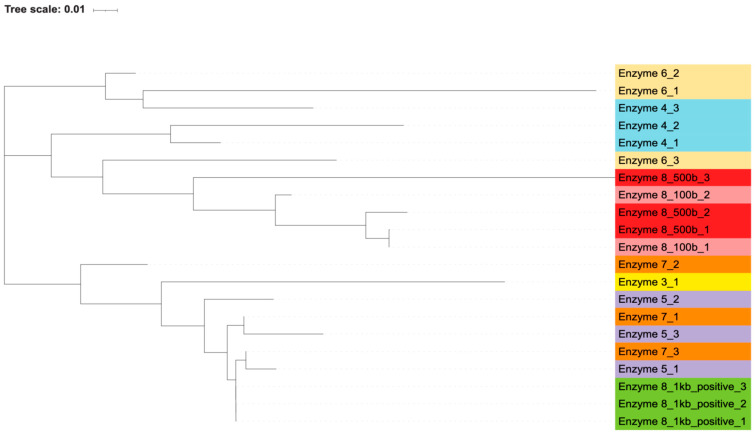
Highest-likelihood phylogeny of reverse sequences. A phylogenetic tree demonstrating the highest-likelihood similarity between contaminating bacterial sequences from various PCR enzymes using the reverse primer for sequencing. Contaminating PCR sequences that occurred in the water control reactions were submitted for Sanger sequencing by the company GENEWIZ. Three sequences from *E. coli* reactions were also included from enzyme 8, as they were double the expected size. All sequences were of the 500 base expected size unless otherwise indicated. As in the tree in Figure 2, the most significant factor in sorting the sequences was the PCR enzyme used for DNA synthesis. This tree was generated in the MEGA11 software package with a maximum likelihood algorithm from an alignment using the Clustal algorithm. A total of 1000 bootstrap replicates were performed. The tree was labeled and visualized using Interactive Tree of Life. Colors are identical for technical replicates of the same enzyme.

By generating phylogenetic trees using the maximum likelihood algorithm, we found that there was an association between the polymerase used and the resulting sequence of the contaminating bands. Most of the polymerases closely associated with themselves (Figure 2 and Figure 3). The exception to this is enzyme 7, which is interspersed with enzymes 3 and 5. Percent identity between sequences, as calculated by the Clustal algorithm, can be found in Table 8. These calculations indicate that while certain enzymes and reactions have highly similar sequences (enzyme 8’s positive reactions), the sequencing results are highly diverse across the different enzymes. The most likely contaminating bacteria based on sequence identity were determined through the use of the NCBI blast algorithm. The likely identities of these bacteria can be found in Supplementary Table S2.

This grouping of polymerases, as well as the distinct sequences that were generated from each contaminant band, indicate that these bacterial DNA contaminants were intrinsic to the polymerase reaction components themselves and not introduced in our own workflow. If the bacterial contamination had been introduced by our handling of the samples, we would expect to see more similar sequences across all the polymerases, without an association between sequences and individual polymerases. Additionally, if the contamination was introduced by us, we would not have expected to see the wide variance in bacterial species and genera that were found via blast search. Furthermore, the contamination of our own reagents or the introduction of contamination during reaction set up would have led to every polymerase reaction being contaminated, but two of the polymerases, enzymes 2 and 9, did not contain apparent bacterial DNA.

## 4. Discussion

It has been previously demonstrated that bacterial DNA contamination can be found in extraction kits, laboratory consumables, and even in PCR reagents [1,2,3,4,5,6,7]. Here, we have extended these previous findings by examining a large cross-section of commercially available PCR enzymes, while previous work has mostly only characterized one enzyme at a time. We have also made a point of detecting and characterizing these contaminants using only endpoint PCR and Sanger sequencing. These methods are attainable even for labs with minimal resources and for whom additional short-read sequencing reads and analysis would be cost- or time-prohibitive.

Here, we demonstrate that not only is DNA contamination widespread in PCR reagents, but also that the dominant bacteria in these reagents varies. This variation indicates that it cannot be assumed that a lab will always have the same contaminating sequences, as this will change with different PCR enzyme selection. The authors would suggest that research groups maintain a single type and lot of enzyme for a set of related experiments. And if the enzyme or lot was to be switched for any reason, then a new set of control experiments should be performed to ensure that there is an accurate depiction of the possible bacterial contaminants. This is shown in these experiments by the difference in contamination profiles of enzyme 1. In previous experiments in our lab, a different lot of this enzyme produced bright bands in all its negative controls of equal size to the V3-4 region of the 16S rRNA (unpublished data). However, in these experiments, there is now only a single reaction of the three that has a faint contamination band, approaching the limit of detection of an agarose gel. This shows how widely the contamination of an enzyme can vary between lots. We would recommend that any PCR enzymes that are used be purchased by specific lots, and that this single lot be maintained for any experiments that will be directly compared to one another.

Importantly, our data show that this contamination of bacterial DNA can be detected with endpoint PCR and Sanger sequencing, two methods which are rapid and accessible for many labs compared to the more expensive and resource-intensive qRT-PCR and short-read sequencing. Microbiome research remains dominated by groups in well-resourced countries, often far from the locations where samples are collected, especially in humans. For these labs, the additional expense and resources required for sequencing negative control reactions is inconsequential. However, many labs are tightly constrained by budgets and may be limited to only being able to sequence a minimum of experimental samples and controls. Our work here is valuable to show that, in these situations, endpoint PCR and Sanger sequencing can be used as valid substitutes for additional short-read sequencing runs.

Together, the data presented here reinforce how important it is to carefully control for bacterial contamination regardless of the analysis methods used. As stated above, bacterial DNA contamination is an ongoing issue in the study of the microbiome, and it is a particular problem in samples that are likely to have a low bacterial burden, as contamination is likely to make up a higher proportion of the DNA present. And even though this is a known issue in the field, it remains underappreciated and, in some cases, uncontrolled for. There are many sources of contamination that should be examined for contamination, such as DNA extraction kits, plastic ware, and as shown here, PCR reagents used for expansion of target sequences. The authors recommend that anytime that a low bacterial burden tissue or environmental sample is going to be examined for bacterial DNA, one of the included control reactions should be used to control for potential contamination of PCR reagents. This control can be used in one of two ways: (1) if the selected PCR enzyme must be used, then the contaminants can be identified and controlled for during analysis; or (2) multiple enzymes and enzyme lots can be tested, and only those that are found to be free of detectable DNA can be selected.

## Figures and Tables

**Table 1:** PCR enzymes utilized in this study.

Enzyme	Manufacturer	Premixed	dNTP Aliquot	Water Aliquot
1	1	No	1	Invitrogen RT-PCR grade
2	1	No	1	Invitrogen RT-PCR grade
3	2	No	1	Invitrogen RT-PCR grade
4	2	No	1	Invitrogen RT-PCR grade
5	3	No	2	Invitrogen RT-PCR grade
6	3	Yes	N/A	Invitrogen RT-PCR grade
7	3	No	2	Invitrogen RT-PCR grade
8	4	No	1	Invitrogen RT-PCR grade
9	5	No	3	Molecular grade water included in kit components

**Table 2:** PCR cycling conditions for enzymes 1 and 2.

Step	Condition	Number of Cycles
Initial Denaturation	95 °C, 2 min	1
Denature	95 °C, 30 s	45
Annealing	55 °C, 30 s	
Elongation	72 °C, 1 min	
Final Elongation	72 °C, 5 min	1

**Table 3:** PCR cycling conditions for enzyme 3.

Step	Condition	Number of Cycles
Initial Denaturation	95 °C, 2 min	1
Denature	95 °C, 30 s	45
Annealing	55 °C, 30 s	
Elongation	68 °C, 1 min	
Final Elongation	68 °C 5, min	1

**Table 4:** PCR cycling conditions for enzyme 4.

Step	Condition	Number of Cycles
Initial Denaturation	98 °C, 2 min	1
Denature	98 °C, 30 s	45
Annealing	60 °C, 30 s	
Elongation	72 °C, 1 min	
Final Elongation	72 °C, 5 min	1

**Table 5:** PCR cycling conditions for enzymes 5, 6, 7.

Step	Condition	Number of Cycles
Initial Denaturation	98 °C, 30 s	1
Denature	98 °C, 10 s	45
Annealing	55 °C, 30 s	
Elongation	72 °C, 30 s	
Final Elongation	72 °C, 5 min	1

**Table 6:** PCR cycling conditions for enzymes 8 and 9.

Step	Condition	Number of Cycles
Initial Denaturation	95 °C, 15 min	1
Denature	94 °C, 30 s	45
Annealing	55 °C, 1 min	
Elongation	72 °C, 1 min	
Final Elongation	72 °C, 10 min	1

**Table 7:** PCR primer sequences.

Primer	Sequence
V3-4 Forward Primer	TCGTCGGCAGCGTCAGATGTGTATAAGAGACAGCCTACGGGNGGCWGCAG
V3-4 Reverse Primer	GTCTCGTGGGCTCGGAGATGTGTATAAGAGACAGGACTACHVGGGTATCTAATCC

**Table 8:** Percent identity of sequencing reads.

6_3	100																			
6_1	57.23	100																		
8_500b_3	50.17	50.35	100																	
4_2	47.72	50.42	57.93	100																
6_2	49.09	55.56	59.03	63.6	100															
8_500b_2	53	54.23	70.2	60.7	67.04	100														
8_100b_1	57.33	59.71	70.32	69.9	73.25	91.97	100													
8_500b_1	57.33	59.71	70.67	69.5	73.73	92.24	99.53	100												
4_3	56.19	60.94	63.23	68.6	77.2	74.09	78.72	79.39	100											
4_1	50.76	56.23	64.31	74.8	69.55	66.2	73.46	73.88	76.22	100										
3_1	55.59	57.89	61.26	65.5	68.63	69.64	77.72	77.48	79.53	74.88	100									
7_2	54.22	59.36	58.46	70.7	71.32	70.83	76.84	76.58	78.55	78.11	77.7	100								
5_2	55.4	57.73	55.36	57.8	67.42	60.87	64.44	64.44	69.77	67.53	73.31	75.91	100							
5_3	54.68	59	62.26	71.2	75.06	69.92	77.54	77.54	79.32	80.18	81.25	89.08	84.24	100						
8_1kb_+_1	52.08	59.19	57.31	67	74.67	70.47	78.71	78.43	82.46	77.95	84.82	85.04	84.15	92.15	100					
8_1kb_+_2	52.13	58.28	55.93	65.2	72.45	68.52	75.94	75.94	79.95	76.13	82.96	82.62	82.39	89.72	98.96	100				
8_1kb_+_3	51.8	58.28	55.93	65.7	72.7	68.8	76.55	76.28	80.3	76.46	83.08	83.25	82.39	90.4	99.48	98.99	100			
7_1	52.38	59.33	57.14	66.2	76.46	70.81	77.27	77.56	81	77.93	83.38	85.6	86.9	93.4	96.51	96.57	96.83	100		
5_1	54.98	60.66	60.8	68	75.41	71.59	78.47	78.71	82.09	78.55	84.88	84.8	86.82	93.29	97.38	95.49	95.45	98.42	100	
7_3	54.34	60.47	59.06	67.7	74.62	71.59	78.63	78.89	82.47	78.47	84.35	85.19	86.97	93.61	97.91	95.74	96.21	98.68	99.27	100
Enzyme	6_3	6_1	8_500b_b3	4_2	6_2	8_500b_2	8_100b_1	8_500b_1	4_3	4_1	3_1	7_2	5_2	5-3	8_1kb_+_1	8_1kb_+_2	8_1kb_+_3	7_1	5_1	7_3

## Data Availability

All sequence information is available for download and inspection from the NCBI GenBank database at the accession numbers found in Supplemental Table S3.

## Supplementary Materials

The following supporting information can be downloaded at: https://www.mdpi.com/article/10.3390/microorganisms13040732/s1, Table S1. Table containing the key for the enzyme and manufacturers used, Table S2. Table containing predicted bacterial identities from blast search, Table S3. Table containing the accession numbers of the sequencing information in the NCBI GenBank database.

## Abbreviations

The following abbreviations are used in this manuscript:

PCR	polymerase chain reaction
qRT-PCR	quantitative real-time polymerase chain reaction
dNTP	deoxynucleotide triphosphate
